# Acute mania with psychotic symptom in post COVID-19 patient

**DOI:** 10.1192/bjo.2021.182

**Published:** 2021-06-18

**Authors:** Sridevi Shanmugam, Praveen Kumar, Blaga Carr

**Affiliations:** 1 Essex Partnership University NHS Foundation Trust; 2 NHS highland

## Abstract

**Aims:**

COVID-19 is an on-going pandemic caused by severe acute respiratory syndrome coronavirus 2 (SARS-CoV-2). Recent evidence suggests that SARS-CoV-2 may be associated with various neuropsychiatric symptoms, including mania. We present a case of a middle aged man presenting with acute mania with psychotic symptoms 20 days post COVID infection in the absence of prior psychiatric illness. This report highlights the need for rigorous neuropsychiatric assessment in patient with symptoms of SARS-CoV-2 infection.

**Method:**

A 52-year-old man of West African origin with past history of hypertension and no previous history of mental health illness presented with acute manic symptoms on background of two weeks of high fever, diarrhoea, mild headache, dry cough and anosmia. He was tested positive for SARS-CoV-2 infection on COVID PCR test. He was under self-isolation along with his family members who exhibited mild symptoms of SARS-CoV-2, none of them required hospital admission. He was initially fearful to seek medical attention but was brought in by family after exhibiting behaviour changes, obsession with toilet cleaning, reckless spending and getting aggressive approximately two weeks after the onset of acute upper respiratory symptoms. He presented elated in mood with pressure of speech and grandiose ideas. Investigations like neuroimaging and bloods were unremarkable. Initial psychiatric assessment found symptoms consistent with acute mania and he was detained under the Mental Health Act. During admission, he was sexually disinhibited and agitated on the ward requiring IM antipsychotics. He was treated with high dose of Olanzapine and Sodium valproate and his symptoms subsided within two weeks.

**Result:**

This case emphasises the manifestation of neuropsychiatric illness post COVID-19 without a background of psychiatric illness, hypoxemia and cerebral infarction.

Based on the CORONERVE Programme and latest retrospective Lancet cohort studies, the period between 14 and 90 days after diagnosis, 5.8% COVID-19 survivors had their first recorded diagnosis of psychiatric illness.

It is also important to consider other organic disease given the simultaneous diagnosis of COVID-19. Although it is not yet possible to confirm here due to the lack of a validated CSF-PCR assay, previous reports have implicated SARS-CoV-2 in the development of viral encephalitis, and this remains an important differential.

**Conclusion:**

Clinicians should be alert to the possibility of patients with COVID-19 developing neuropsychiatric complications post SARS-CoV-2 infection, mandating the need for vigilant initial neuropsychiatric assessment and possibly follow-up care in 3 months.

